# DNA Synthesis during Endomitosis Is Stimulated by Insulin via the PI3K/Akt and TOR Signaling Pathways in the Silk Gland Cells of *Bombyx mori*

**DOI:** 10.3390/ijms16036266

**Published:** 2015-03-18

**Authors:** Yaofeng Li, Xiangyun Chen, Xiaofang Tang, Chundong Zhang, La Wang, Peng Chen, Minhui Pan, Cheng Lu

**Affiliations:** 1 State Key Laboratory of Silkworm Genome Biology, Southwest University, Chongqing 400716, China; E-Mails: lyfengcxy2010@163.com (Y.L.); chenxyunxy@163.com (X.C.); txf0113@163.com (X.T.); zcd308@163.com (C.Z.); wangla666@163.com (L.W.); chenpengju1122@126.com (P.C.); 2 Key Laboratory for Sericulture Functional Genomics and Biotechnology of Agricultural Ministry, Southwest University, Chongqing 400716, China; 3 Department of Biochemistry and Molecular Biology, Chongqing Medical University, Chongqing 400016, China

**Keywords:** *Bombyx mori*, silk gland, DNA synthesis, insulin signaling

## Abstract

Silk gland cells undergo multiple endomitotic cell cycles during silkworm larval ontogeny. Our previous study demonstrated that feeding is required for continued endomitosis in the silk gland cells of silkworm larvae. Furthermore, the insulin signaling pathway is closely related to nutritional signals. To investigate whether the insulin signaling pathway is involved in endomitosis in silk gland cells, in this study, we initially analyzed the effects of bovine insulin on DNA synthesis in endomitotic silk gland cells using 5-bromo-2'-deoxyuridine (BrdU) labeling technology, and found that bovine insulin can stimulate DNA synthesis. Insulin signal transduction is mainly mediated via phosphoinositide 3-kinase (PI3K)/Akt, the target of rapamycin (TOR) and the extracellular signal-regulated kinase (ERK) pathways in vertebrates. We ascertained that these three pathways are involved in DNA synthesis in endomitotic silk gland cells using specific inhibitors against each pathway. Moreover, we investigated whether these three pathways are involved in insulin-stimulated DNA synthesis in endomitotic silk gland cells, and found that the PI3K/Akt and TOR pathways, but not the ERK pathway, are involved in this process. These results provide an important theoretical foundation for the further investigations of the mechanism underlying efficient endomitosis in silk gland cells.

## 1. Introduction

The silk glands of silkworm larvae, which synthesize and secrete silk protein, can be divided into three anatomically and physiologically distinct regions: the anterior silk gland (ASG), middle silk gland (MSG) and posterior silk gland (PSG). The silk gland is formed in the embryonic stage and contains approximately 1080 cells. These cells undergo multiple endomitotic cell cycles during silkworm larval ontogeny, producing the vast increase (approximately 400,000 times) in the haploid genomic content per nuclei [1]. The DNA synthesis is highly efficient in living organisms. Elucidation of the mechanism of endoreplication in silk gland cells is, therefore, of biological significance.

Our previous study indicated that the DNA synthesis activity of endomitotic silk gland cells is dependent on nutritional status [2]. The insulin/PI3-kinase pathway and the target of rapamycin (TOR) pathway are closely regulated by nutritional signals [3], and increasing the PI3K signaling or TOR signaling can bypass the nutritional requirement and induce endocycle progression in *Drosophila melanogaster* [4]. The involvement of insulin signaling in activation of the endocycle has been confirmed in several studies [5,6,7,8,9]. Furthermore, recent studies have shown that the key genes of the insulin signal transduction pathway and the TOR signaling pathway are also expressed in the silk gland [10,11]. However, the involvement of insulin signaling in the activation of endomitotic DNA synthesis in the silk gland cells remains to be determined.

In *Drosophila melanogaster*, the insulin signaling pathway is highly conserved [12,13]. Following ligation by insulin, the insulin receptor is phosphorylated leading to the activation of a phosphorylation cascade via the insulin receptor substrate protein chico, and subsequent activation of phosphoinositide 3-kinase (PI3K) [14,15]. The catalytic subunit of PI3K p110 phosphorylates the lipid phosphatidylinositol-4,5-P(2) (PIP2) to generate phosphatidylinositol-3,4,5-P(3) (PIP3), which acts as a second messenger in the activation of Akt. Subsequently, Akt activates the target of rapamycin complex 1 (TORC1), which has two key downstream targets: p70 ribosomal protein S6 kinase (S6K) and translational repressor 4E-binding protein (4E-BP) [16]. The TOR pathway plays an important role in cellular processes such as protein translation, cell cycle progression and growth [3]. In vertebrates, the insulin receptor also transmits signals to the extracellular signal-regulated kinase (ERK) pathway [17].

Bombyxin is a structurally insulin-related peptide of the silkworm, *Bombyx mori* [18,19]. Bombyxin genes are expressed predominantly in the brain and at low levels in the ganglia, epidermis, testis, ovary, fat body, silk gland, malpighian tubule, midgut and hindgut [20,21,22]. Nevertheless, it is difficult to obtain the native insulin-like bombyxin peptides. Moreover, it has been demonstrated that bovine insulin can stimulate DNA synthesis in the prothoracic gland [23] and promotes cell proliferation in the larval hematopoietic organ of the silkworm [24]. In addition to this, bovine insulin has been shown to act as a substitute for bombyxin-II, the insulin-like peptide in *B. mori*, together with hemolymph factor(s) to stimulate mitotic division of granulocytes [25]. Therefore, we used bovine insulin as a substitute for bombyxin and investigated the effects of bovine insulin on DNA synthesis in silkworm silk gland cells. Our results showed that bovine insulin activated endomitotic DNA synthesis in silk gland cells and the PI3K/Akt and TOR/S6K/4E-BP pathways, but not the ERK pathway, were involved in this process.

## 2. Results

### 2.1. Insulin Activated Endomitotic DNA Synthesis of Silk Gland Cells

**Figure 1:**
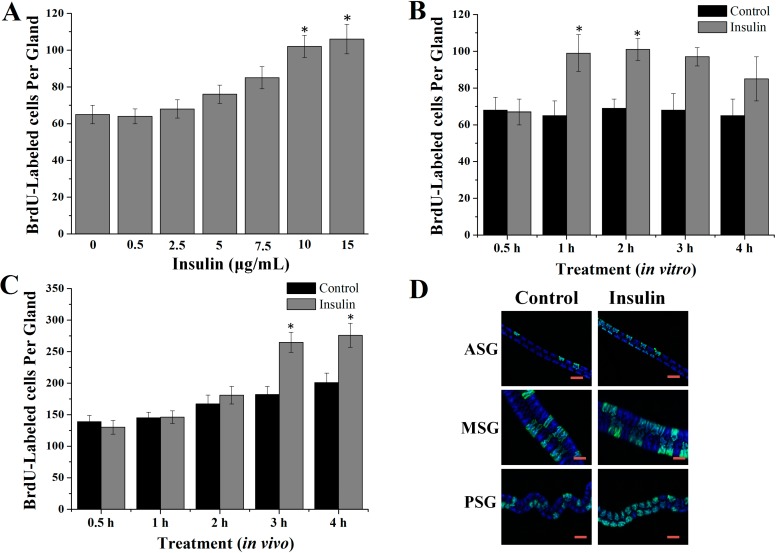
Effects of insulin on DNA synthesis in silk gland cells. (**A**) Concentration dependent effects *in vitro*; (**B**) Time-dependent effects *in vitro*; (**C**) Time-dependent effects *in vivo*; (**D**) Representative images of BrdU-labeled cells in the silk gland cells at 3 h after the injection of insulin. Scale bar: 100 µm. ASG: anterior silk gland; MSG: middle silk gland; PSG: posterior silk gland. * p < 0.05.

We first investigated the effect of insulin on endomitotic DNA synthesis in endomitotic silk gland cells *in vitro*. A dose-dependent increase in endomitotic DNA synthesis was observed in the silk gland cells incubated for 1 h in the presence of insulin at concentrations exceeding 5 µg/mL, with the effects reaching the level of statistical significance at concentrations exceeding 10 µg/mL (*p <* 0.05) (Figure 1A). No increase in the number of BrdU-labeled silk gland cells was observed following incubation with 1.74 μM bovine insulin for 0.5 h, while a significant increase was observed at 1 h and maintained to 2 h, followed by a slight decline (*p <* 0.05) (Figure 1B).

The effects of insulin on DNA synthesis in silk gland cells were then investigated *in vivo* by injection of insulin into day 1 fourth instar larvae. Compared with the controls, a significant increase (45.6%) in the number of BrdU-labeled cells in the glands was observed after 3 h (*p <* 0.05) (Figure 1C,D).

### 2.2. Effects of Specific Inhibitors LY294002, Rapamycin and U0126 on DNA Synthesis of Silk Gland Cells

**Figure 2:**
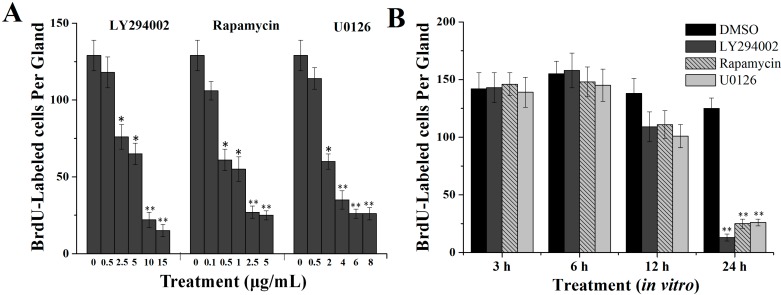

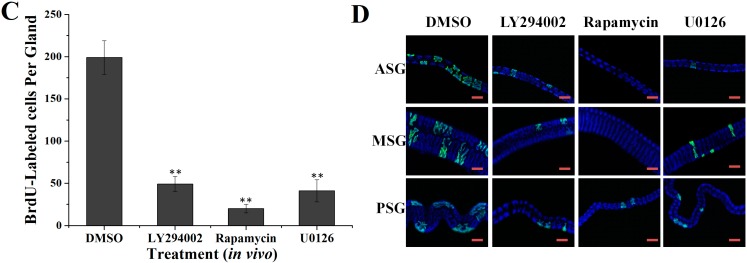
Effects of the specific pathway inhibitors on DNA synthesis in silk gland cells. (**A**) Concentration dependent effects *in vitro*; (**B**) Time-dependent effects *in vitro*; (**C**) Effects of inhibitors *in vivo*; (**D**) Representative images of BrdU-labeled silk gland cells 24 h after injection with each specific inhibitor. Scale bar = 100 µm. ASG: anterior silk gland; MSG: middle silk gland; PSG: posterior silk gland. * p < 0.05, ** *p* < 0.01.

PI3K, TOR and ERK are important proteins in insulin receptor signaling in vertebrates [7,26,27]. To determine the involvement of these pathways in endomitotic DNA synthesis in silk glands cells, we examined the effect of specific inhibitors of PI3K (LY294002) [28], TORC1 (rapamycin) [29] and ERK kinase (MEK) (U0126) [30] both *in vitro* and *in vivo*. The silk glands were dissected and then cultured in control medium with or without each specific inhibitor. All three inhibitors mediated dose-dependent decreases in endomitotic DNA synthesis in silk glands cells *in vitro* (Figure 2A). In the presence of LY294002, the inhibitory effect was significant (*p <* 0.05) at 5 μg/mL, and very significant (*p* < 0.01) at 10 and 15 μg/mL. In the presence of rapamycin, the inhibitory effect was significant (*p <* 0.05) 0.5 and 1 µg/mL, and very significant (*p* < 0.01) at 2.5 and 5 μg/mL. In the presence of U0126, this effect was significant (*p <* 0.05) at 2 µg/mL, and very significant (*p* < 0.01) at 4, 6 and 8 μg/mL. As shown in Figure 2B, compared to those incubated in control medium, very significant (*p* < 0.01) reductions in the number of BrdU-labeled silk gland cells were observed at 24 h following incubation with medium containing specific inhibitors LY22934 (89%), rapamycin (79%) and U0126 (78.5%).

The involvement of PI3K, TOR and ERK pathways in DNA synthesis in silk gland cells were then investigated *in vivo* by injection of each specific inhibitor LY294002, rapamycin or U0126 into day 1 fourth instar larvae. After 24 h, endomitotic DNA synthesis of the silk gland cells was very significantly (*p* < 0.01) inhibited (Figure 2C,D). Compared with the controls, the BrdU-positive cell count was reduced by 75% by LY294002, 89.9% by rapamycin and 79.4% by U0126.

### 2.3. Involvement of the PI3K/Akt and TOR Signaling Pathway in Insulin-Stimulated Endomitotic DNA Synthesis

**Figure 3:**
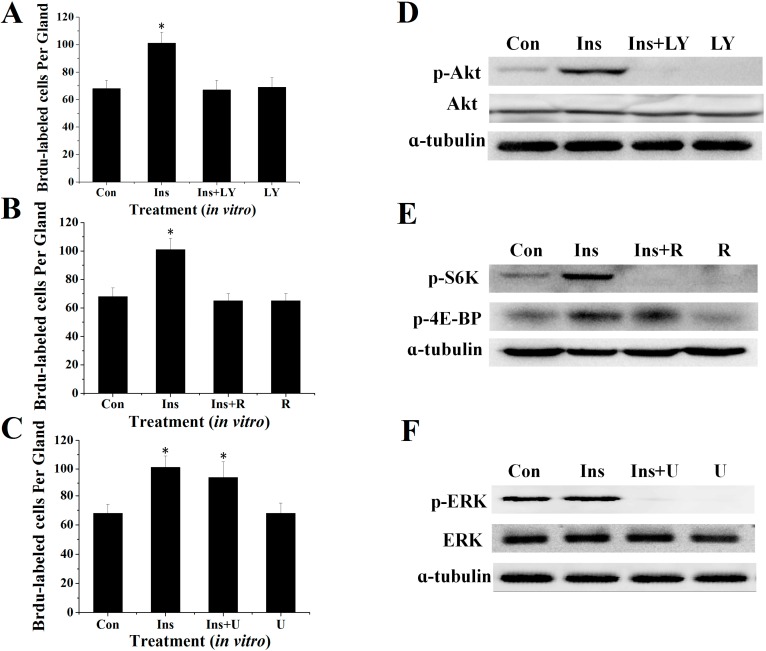
Insulin-activated DNA synthesis of silk gland cells is dependent on PI3K/Akt and TOR signaling, but not ERK signaling. The silk glands of day 1 fourth instar larvae were preincubated with LY294002 (48.8 μM), rapamycin (5.5 μM) or U0126 (21 μM) for 45 min and then incubated with medium containing insulin (1.74 μM) with or without LY294002 (48.8 μM) (**A**), rapamycin (5.5 μM) (**B**) or U0126 (21 μM) (**C**) for 1 h. The silk glands were labeled with BrdU and then analyzed. After preincubation for 45 min, the silk glands were incubated with medium containing 1.74 μM insulin with or without LY294002 (**D**), rapamycin (**E**) or U0126 (**F**) for 15 min. Silk gland lysates were prepared and subjected to an immunoblot analysis with their corresponding antibodies. The results shown are representative of three independent experiments. Con: control; Ins: insulin; LY: LY294002; R: rapamycin; U: U0126. * p < 0.05.

The involvement of the PI3K, TOR or ERK signaling pathways in the mechanism by which insulin activates DNA synthesis of silk gland cells was analyzed by investigating the effects of the specific inhibitors of PI3K (LY294002), MEK (U0126) or TORC1 (rapamycin) on the DNA synthesis induced by insulin *in vitro*. The PI3K inhibitor (LY294002) and the TORC1 inhibitor (rapamycin) could inhibit the stimulatory effects of the endomitotic DNA synthesis by insulin (Figure 3A,B). Surprisingly, the MEK inhibitor (U0126) could not obviously inhibit the activation by the insulin, with only a slight decrease in the number of BrdU-positive cells observed (Figure 3C).

We then analyzed the effects of these treatments on protein phosphorylation by Western blotting. Western blot analysis showed that the phosphorylation of Akt was increased in the silk glands by bovine insulin. In the presence of the PI3K inhibitor LY294002, bovine insulin-stimulated phosphorylation of Akt was greatly inhibited (Figure 3D). Similarly, S6K and 4E-BP phosphorylation was increased in the presence of bovine insulin compared to that in the control medium. Moreover, rapamycin inhibited bovine insulin-stimulated S6K phosphorylation. In contrast, the insulin-stimulated 4E-BP phosphorylation was not reduced by rapamycin (Figure 3E). The level of phosphorylated ERK was not dramatically changed by insulin (Figure 3F).

### 2.4. PI3K/Akt Signaling Is an Upstream Signaling Pathway for Insulin-Activated TOR Signaling

**Figure 4:**
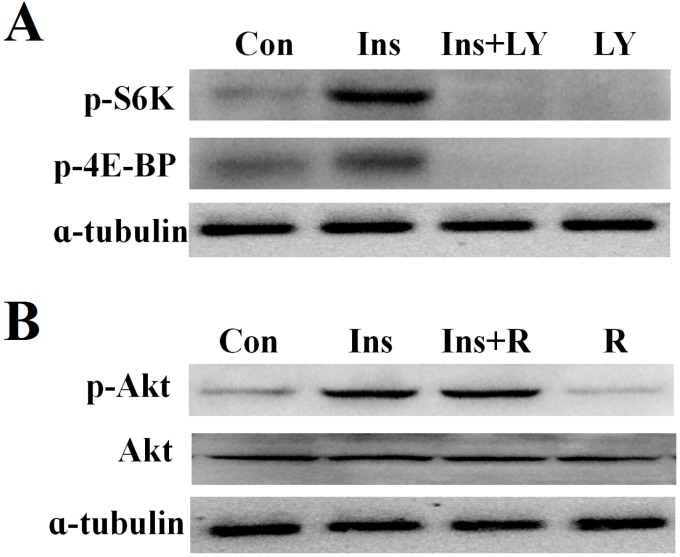
The relationship of PI3K and TOR signaling in silk gland cells. (**A**) Effects of insulin-stimulated S6K and 4E-BP phosphorylation by LY294002; and (**B**) Effects of insulin-stimulated Akt phosphorylation by rapamycin. The results shown are representative of three independent experiments.

The relationship between PI3K and TOR signaling was also investigated in the silk glands in the above-described manner. Western blot analysis showed that the PI3K inhibitor, LY294002, reduced levels of insulin-induced S6K and 4E-BP phosphorylation (Figure 4A). To determine whether TORC1 functions upstream or downstream of Akt, we examined the effects of rapamycin on the insulin-induced phosphorylation of Akt. The results showed that rapamycin did not inhibit Akt phosphorylation (Figure 4B).

## 3. Discussion

Our present study clearly showed that the DNA synthesis of the endomitotic silk gland cells was inhibited in a time- and dose-dependent manner by the specific inhibitors of the insulin signaling pathway in vertebrates (LY294002, rapamycin or U0126). Furthermore, insulin stimulated DNA synthesis via the PI3K/Akt and TOR pathways. These results indicate that the endomitotic DNA replication in silk glands cells is, at least in part, regulated via the insulin signaling pathway. Numerous studies have been performed regarding the mechanism of endomitosis in the salivary glands of *D. melanogaster*, which is the homologous organ of the silk gland of *B. mori*. These studies have also shown that the PI3K/Akt pathway and TOR pathway are involved in the regulation of endoreplication. Inhibiting PI3K activity by overexpressing the dPI3K adaptor subunit p60 or a negative regulator of insulin signaling phosphatase PTEN leads to decreased cell size and small nuclei with far less DNA than adjacent control cells [3]. Furthermore, ectopic expression of Dp110 in the developing salivary gland causes tissue overgrowth [31], while the salivary glands in the *chico*, *dS6K* or *dTOR* mutants are similar in size [32,33].

Bovine insulin activated DNA synthesis in silk gland cells, indicating that insulin signaling plays a role in the silk gland development; however, the time required for insulin-induced activation of DNA synthesis in the silk gland cells *in vitro* was different to that observed *in vivo*. It can be speculated that insulin acts directly on silk glands *in vitro*, whereas the stimulatory signal is dispersed to other tissues during the process of transport to the silk glands that is required *in vivo*.

**Figure 5:**
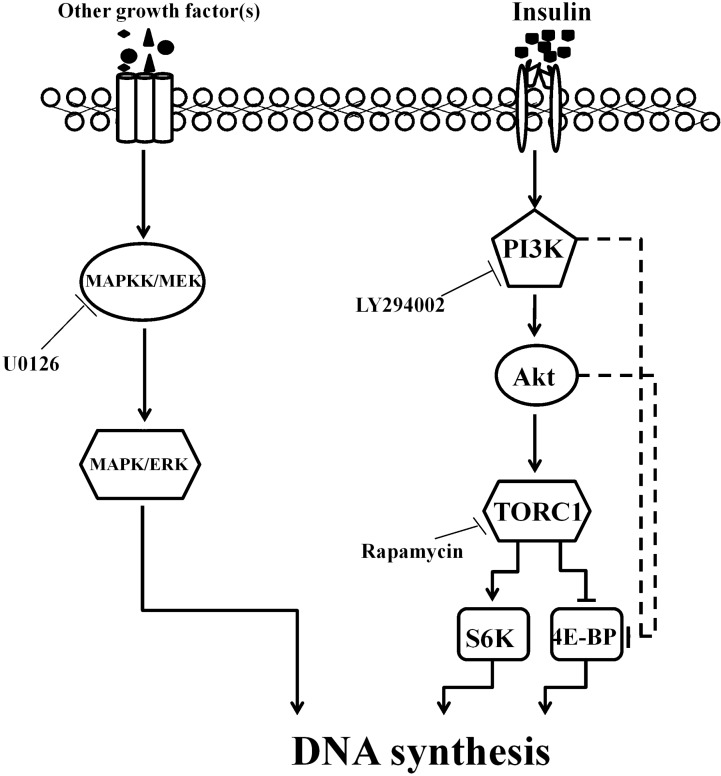
A predicted network that links the growth factor signaling pathways with silk gland cell DNA synthesis in the silkworm. Insulin activated silk gland DNA synthesis via PI3K/AKT/TOR signaling. MAPK/ERK signaling may be activated by other growth factor(s). Solid lines indicate demonstrable or highly likely relations; dashed lines indicate hypothetical interactions.

In our study, bovine insulin stimulated the phosphorylation of Akt, S6K and 4E-BP in the silk glands, which indicated that both PI3K/Akt and TOR signaling are involved in insulin-stimulated DNA synthesis. PI3K/Akt signaling is also activated by insulin in the prothoracic glands of the silkworm [23] and the ovaries of *Aedes aegypti* [34]. Additionally, the PI3K inhibitor LY294002 abolished the insulin-stimulated S6K and 4E-BP phosphorylation, while the TORC1 inhibitor rapamycin did not inhibit Akt phosphorylation, indicating that PI3K is an upstream effector of TORC1. Both the PI3K inhibitor LY294002 and the TORC1 inhibitor rapamycin could inhibit insulin-stimulated DNA synthesis, indicating that PI3K and TOR pathways operate cooperatively in the delivery of the insulin signal. Previous studies have demonstrated that the PI3K and TOR pathways are highly conserved between *D. melanogaster* and higher eukaryotes, which are the most important target pathways in insulin signaling and the PI3K/Akt pathway lies upstream of TORC1 [35,36,37]. S6K and 4E-BP are two important targets of TORC1. Surprisingly, the inhibitory effect of rapamycin on insulin-stimulated phosphorylation of 4E-BP was not detected, while insulin-stimulated 4E-BP phosphorylation was blocked by the PI3K inhibitor, LY294002, in our study. Previous studies showed that LY294002 is a broad-specificity PI3K inhibitor and also inhibits the kinase activity of mammalian target of rapamycin (mTOR) [38,39,40]. The insulin-stimulated 4E-BP phosphorylation was blocked by LY294002 in this study, which could be because LY294002 inhibits the kinase activity of PI3K and TOR in the silk gland. In *D. melanogaster* Schneider 2 cells, 4E-BP phosphorylation is also regulated through PI3K and Akt [41]. Thus, we suspect that insulin-stimulated 4E-BP phosphorylation is also dependent in a PI3K- and Akt-dependent manner in the silk gland cells (Figure 5).

In vertebrates, the pleiotropic effects of insulin are controlled, in part, by the PI3K signaling pathway and ERK signaling pathway [17,42,43]. Our results indicate that the ERK pathway is not involved in the mechanism by which insulin stimulates DNA synthesis in silk gland cells, although it is involved in endomitotic DNA synthesis. It has also been reported that insulin does not stimulate ERK signaling in the prothoracic glands [23]. There are no reports that the ERK signaling pathway plays a role in insulin signaling in insects. The ERK pathway appears to be activated by other ligands and receptor tyrosine kinases. Thus, the regulatory mechanism of endomitosis in silk gland cells is complicated, and the growth of silk glands is also likely controlled by other growth factor(s) (Figure 5).

Here, we hypothesize that insulin can increase the supply of nutrients, promote cytoplasmic growth and protein synthesis, and initiate the PI3K/Akt/TOR pathway to stimulate DNA synthesis. A previous study showed that bombyxin, an insect insulin-related peptide, reduced the level of carbohydrate in the hemolymph of *Bombyx mori* and facilitated its transport into tissues [44]. The ectopic expression of PI3K in fat body cells increased the opacity of the cytoplasm and promoted nutrient storage [4]. It has long been known that mTORC1 enhances cell growth by promoting ribosome biogenesis and mRNA translation [3]. Moreover, studies on mitotic systems suggest that the PI3K/Akt/TOR pathway influences the accumulation of Cyclin D and Cyclin E at the protein level via regulation of translation or turnover [45]. The levels of Cyclin E or Cyclin E/Cdk2 kinase activity oscillate during the endocycle in *Drosophila* [46]. However, their interactions with the growth stimulating pathways are unclear. The silk gland is not only a spinning organ, but also a good model for the study of endomitosis. Thus, the findings of the current study lay a foundation for future research in the regulatory mechanisms of the endoreplication in silk gland cells.

## 4. Experimental Section

### 4.1. Experimental Animals

Larvae of the *B. mori* strain 09-030 were obtained from the Silkworm Gene Bank of Southwest University, Chong Qing, China and were reared on fresh mulberry leaves at 25 °C under a 12 light: 12 dark photoperiod. The animal protocols used in this work were evaluated and approved by the Animal Ethics Committees of Southwest University.

### 4.2. Reagents and Antibodies

Grace’s insect cell culture medium was purchased from Gibco (Grand Island, NY, USA). Bovine insulin was purchased from Sigma (St. Louis, MO, USA). The PI3K inhibitor (LY294002) was purchased from Sigma. The mitogen-activated protein kinase (MAPK)/ERK kinase (MEK) inhibitor (U0126) and the TORC1 inhibitor (rapamycin) were purchased from Calbiochem (San Diego, CA, USA). Protease and phosphatase inhibitors were purchased from Roche (Roche, Basel, Switzerland). The *In Situ* Cell Proliferation kit was purchased from Roche.

Antibodies directed against Drosophila phosphorylated Akt (Ser505) and anti-total-Akt, phospho-ERK (Thr202/Tyr204), anti-total-ERK, phospho-4E-BP1 (Thr37/46), and anti-α-tubulin were purchased from Cell Signaling Technology (Beverly, MA, USA). Anti-phospho-S6K (Thr412) was purchased from Upstate (Lake Placid, NY, USA). The horseradish peroxidase (HRP) conjugated goat anti-rabbit IgG antibody was purchased from Cell Signaling Technology.

### 4.3. In Vitro Culture of Silk Glands

Day 1 fourth instar larvae were surface sterilized in 75% ethanol for 2–3 min, rinsed in sterile water and placed on ice. The silk glands were dissected and rinsed three times in lepidopteran saline (110 mM KCl; 15 mM MgCl_2_; 4 mM NaCl; 4 mM CaCl_2_), and three times in Grace’s insect cell culture medium. Subsequently, the silk glands were maintained in 500 µL of basal medium (Grace’s insect cell culture medium supplemented with 2% FBS) at 27 °C.

### 4.4. Effects of Insulin on DNA Synthesis in Silk Gland Cells

#### 4.4.1. Dose-Dependent Effects *in Vitro*

Silk glands from day 1 fourth instar larvae were incubated in basal medium (control) or medium containing different concentrations (0, 0.5, 2.5, 5, 7.5, 10 and 15 µg/mL) of bovine insulin for 1 h. After incubation, the silk glands were labeled with BrdU, and the number of the BrdU-labeled cells was determined.

#### 4.4.2. Time-Dependent Effects *in Vitro*

Silk glands of day 1 fourth instar larvae were incubated in basal medium (control) or medium containing 1.74 μM bovine insulin for different time-periods (0.5, 1, 2, 3, and 4 h). After incubation, the silk glands were labeled with BrdU, and the number of the BrdU-labeled cells was determined.

#### 4.4.3. Effects of the Insulin *in Vivo*

Bovine insulin (10 μg) was injected into day 1 fourth instar larvae. The silk glands were dissected and labeled with BrdU at different time-points (0.5, 1, 2, 3, and 4 h), and the number of the BrdU-labeled cells was determined.

### 4.5. Effects of the Specific Inhibitors on DNA Synthesis in Silk Gland Cells

#### 4.5.1. Dose-Dependent Effects *in Vitro*

Silk glands from day 1 fourth instar larvae were incubated in control medium or medium containing different concentrations of LY294002, rapamycin or U0126. After 24 h, the silk glands were labeled with BrdU, and the number of the BrdU-labeled cells was determined.

#### 4.5.2. Time-Dependent Effects *in Vitro*

The silk glands of day 1 fourth instar larvae were incubated in control medium or medium containing LY294002 (48.8 μM), rapamycin (5.5 μM) or U0126 (21 μM). After incubation for different time-periods (3, 6, 12 and 24 h), the silk glands were labeled with BrdU, and the number of the BrdU-labeled cells was determined.

#### 4.5.3. Effects of Inhibitors *in Vivo*

Each specific inhibitor LY294002 (15 μg), rapamycin (5 µg) or U0126 (8 µg) was injected into day 1 fourth instar larvae. The silk glands were dissected, and the number of the BrdU-labeled cells was determined at 24 h.

### 4.6. BrdU Incorporation and Immunocytochemical Staining

Proliferating silk gland cells were labeled with 5'-bromo-2'-deoxyuridine (BrdU) *in vitro* using a previously described method [2]. BrdU was identified by a specific monoclonal antibody [47]. The total number of BrdU-labeled cells per silk gland was counted directly under an Olympus BX21 microscope (Olympus, Tokyo, Japan).

### 4.7. Western Blot Analysis

The silk glands of day 1 fourth instar larvae were preincubated with LY294002 (48.8 μM), rapamycin (5.5 μM) or U0126 (21 μM) for 45 min, and then incubated with medium containing 1.74 μM insulin with or without each inhibitor LY294002 (48.8 μM), rapamycin (5.5 μM) or U0126 (21 μM) for 15 min. *In vitro* cultured silk glands were then homogenized in sodium dodecyl sulfate (SDS) lysis buffer (Beyotime Biotechnology, Zhejiang, China) containing protease and phosphatase inhibitors. Lysates were boiled in an equal volume of SDS-polyacrylamide gel electrophoresis (SDS-PAGE) sample loading buffer (Beyotime Biotechnology, China) for 10 min. Proteins were subjected to SDS-PAGE (10% or 12% gel). Following electrophoresis, transferred to PVDF membranes and blocked with 5% (*w*/*v*) non-fat dried milk in Tris-buffered saline containing 0.1% Tween 20 (TBST) at room temperature for 1 h. Membranes were then incubated (overnight at 4 °C with gentle agitation) in the presence of primary detection antibody diluted in 10 ml TBST containing 5% (*w*/*v*) bovine serum albumin. The commercial antibody directed against phosphorylated Drosophila Akt (Ser505) [23] and anti-total-Akt [48], phospho-4E-BP1 (Thr37/46) [49], phospho-S6K (Thr412) [50], phospho-ERK (Thr202/Tyr204) and total-ERK antibodies [51], as those previously reported were used to detect PI3K signaling, TOR signaling and MAPK/ERK signaling. In addition, we detected many contaminating non-specific bands by immunoblotting *B. mori* silk gland lysates with commercially available anti-total 4E-BP and anti-total S6K antibodies, so we used the anti-ɑ-tubulin antibody as loading controls. Subsequently, membranes were incubated (1 h at room temperature with gentle agitation) with HRP-conjugated goad anti-rabbit secondary antibody diluted in blocking buffer.

### 4.8. Statistical Analysis

The results are the means ± SEM (*n* = 10). Student’s *t*-tests were carried out for statistical analysis (* p < 0.05, ** *p* < 0.01).
